# Properties of Halide Perovskite Photodetectors with Little Rubidium Incorporation

**DOI:** 10.3390/nano12010157

**Published:** 2022-01-03

**Authors:** Yuan-Wen Hsiao, Jyun-You Song, Hsuan-Ta Wu, Ching-Chich Leu, Chuan-Feng Shih

**Affiliations:** 1 Department of Electrical Engineering, National Cheng Kung University, Tainan 70101, Taiwan; n28064012@mail.ncku.edu.tw (Y.-W.H.); c2781130@gmail.com (J.-Y.S.); 2 Department and Institute of Electrical Engineering, Minghsin University of Science and Technology, Hsinchu 30401, Taiwan; htwu@must.edu.tw; 3 Department of Chemical and Materials Engineering, National University of Kaohsiung, Kaohsiung 81148, Taiwan; 4 Hierarchical Green-Energy Materials (Hi-GEM) Research Center, National Cheng Kung University, Tainan 70101, Taiwan

**Keywords:** Rb doping, perovskite, photodetector, detectivity

## Abstract

This study investigates the effects of Rb doping on the Rb-formamidinium-methylammonium-PbI_3_ based perovskite photodetectors. Rb was incorporated in the perovskite films with different contents, and the corresponding photo-response properties were studied. Doping of few Rb (~2.5%) was found to greatly increase the grain size and the absorbance of the perovskite. However, when the Rb content was greater than 2.5%, clustering of the Rb-rich phases emerged, the band gap decreased, and additional absorption band edge was found. The excess Rb-rich phases were the main cause that degraded the performance of the photodetectors. By space charge limit current analyses, the Rb was found to passivate the defects in the perovskite, lowering the leakage current and reducing the trap densities of carriers. This fact was used to explain the increase in the detectivity. To clarify the effect of Rb, the photovoltaic properties were measured. Similarly, h perovskite with 2.5% Rb doping increased the short-circuit current, revealing the decline of the internal defects. The 2.5% Rb doped photodetector showed the best performance with responsivity of 0.28 AW^−1^ and ~50% quantum efficiency. Detectivity as high as 4.6 × 10^11^ Jones was obtained, owing to the improved crystallinity and reduced defects.

## 1. Introduction

Organic perovskites have become the most important candidate in the future of high-efficiency solar cells because of easy fabrication, high absorption coefficient, wide band gap range, and fact charge transport [1]. As a hybrid organic-inorganic material, the crystalline properties and direct band gap characteristics mean that perovskites have different optoelectronic properties to the inorganic materials. The halide perovskites are defined by the formula AMX_3_ [2], which is composed by a monovalent cation, A = formamidinium (H_2_NCHNH_2_^+^, FA) and methylammonium (CH_3_NH_3_^+^, MA); a divalent metal M from group II; and a halide anion X [3]. When the octahedral anions in the A sites were replaced by the inorganic cations such as cesium (Cs^+^) and rubium (Rb^+^), the hybrid metal halide perovskites became more stable [4]. Various successful reports have used the alkaline stabilized perovskite for solar cells [5], lasers [6], and light-emitting diodes applications [7].

Recently, perovskite photodetectors (PDs) have been shown to have potential for light detection from visible to near infrared. Adjusting the structure by A-site doping improves the overall performance of the photodetector, resulting in high responsivity, low noise and fast response. For example, MA lead-halide-based PDs exhibited high detectivity, low noise, and large linear dynamic range. However, it is very unstable at high temperature. Additionally, the quantum efficiency of the (MA^+^) perovskite based PDs is low [8,9,10,11]. FA^+^ perovskite improves the thermal stability, but the yellow δ-phase usually emerged that seriously degrades the properties. Stability of the perovskite can be improved by using mixing cations [12]. These kind of devices have better performance than the single and double-cation ones and better stability [13,14,15]. For example, we have demonstrated the FA-MA-Cs mixed perovskite PDs have superior behaviors than the FA-MA PDs [16]. Snaith et al. have reported that the FA-Cs mixed cationic perovskite film can be stable at a temperature above 100 °C [17]. Rb is also an element of group I, which has been reported to reduce the non-perovskite phase [18,19,20]. To our best knowledge, the influences of Rb doping on the performance of FAMAPbI_3_ PDs has not been studied. In this work, the Rb-doped organic perovskite PDs were investigated. The Rb-doped PDs demonstrated a wide spectrum coverage in the visible range that has a maximum external quantum efficiency (EQE) of ~50%.

## 2. Materials and Methods

Rb_x_(FA_0.75_MA_0.25_)_1-x_PbI_3_ precursor solution was made by dissolving methylammonium (MA) iodide, formamidinium (FA) iodide, and lead iodide with 0.8 mL dimethylformamide (DMF) and 0.2 mL of dimethyl sulfoxide. The concentration of the perovskite precursor solution was 1.2 M. The solution was stirred until it was completely dissolved to form FA_0.75_MA_0.25_PbI_3_ perovskite precursor solution. Rubidium iodide was pre-dissolved in dimethyl sulfoxide with a molar concentration of 1.5 M and then added to form the Rb_x_ (FA_0.75_MA_0.25_)_1-x_ perovskite precursor, in which x = 0, 0.025, 0.05, and 0.075. The indium tin oxide (ITO) glass was treated by acetone, isopropyl alcohol, and distilled water before coating. Then, it was dried with nitrogen and ultraviolet light ozone treated for 20 min. The perovskite precursor solution was spin-casted on ITO substrate. 200 μL anti-solvents of chlorobenzene (CB) was dropped at the last 15 s, and then annealed at 50 °C for 20 min then heated up to 100 °C for 1 h.

The stacking sequence of the photodetector was ITO/PEDOT:PSS/perovskite/PC_61_BM/BCP/Al. The PEDOT:PSS layer was coated on ITO-glass firstly. The perovskite thin film was spin-coated on the PEDOT:PSS layer by one-step method. The PC_61_BM([6,6]-phenyl C61 butyric acid methyl ester) solutions was prepared by dissolving 20 mg PC_61_BM powder in 1 mL chlorobenzene, continuously stirring for >12 h to ensure the full dissolution. PC_61_BM solution was spin-coated at 3000 rpm for 40 s. Bathocuproine (BCP) was coated on the PCBM as the buffer layer. A 150 nm-thick Al electrode was thermally evaporated using a shadow mask. The structure of the PD device was plotted in Figure S1.

The microstructure and elementary mapping of the perovskite thin films were analyzed by scanning electron microscope (SEM, Hitachi SU8000, Tokyo, Japan) with energy dispersive X-ray spectroscopy (EDS, Bruker X-flash FlatQuad 5060FQ, Berlin, Germany). The crystalline structures were characterized by X-ray diffraction (XRD, Bruker D8 Discover, Karlsruhe, Germany). The steady-state photoluminescence (PL) spectra were collected by Horiba Jobin Yvon LabRAM HR system (Kyoto, Japan). The absorption spectrum of perovskite thin film was collected by using a HITACHI U4100 UV–vis–NIR spectrometer (Tokyo, Japan). The space-charge limit current (SCLC) was analyzed by current-voltage (I–V) measurement using Agilent E5270B in the dark. White light-emitting diode(300 lm, 10 Hz) was used to measure the photo-response, and solar simulator (AM 1.5, 100 mW/cm^2^) was used to measure the solar cells. The photocurrent was acquired using a Tektronix TBS-1104 digital oscilloscope (Beaverton, OR, USA).

## 3. Results

**Figure 1:**
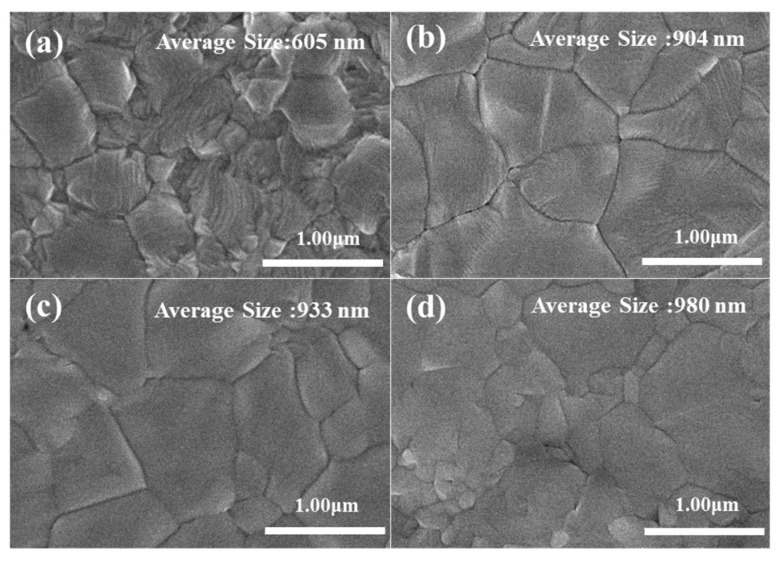
Top-view SEM morphology of Rb-doped (**a**) 0%, (**b**) 2.5%, (**c**) 5%, (**d**) 7.5% perovskite films.

Figure 1a–d shows the morphologies of the perovskite films with 0%, 2.5%, 5% and 7.5% Rb, respectively. The grain size was estimated in the insets, increasing with the Rb content. Besides, the grain size distribution was much improved by 2.5–5% Rb doping, as compared with the pure (FA_0.75_MA_0.25_)_1-x_PbI_3_ film that small crystallites embedded. When the Rb content was increased to 7.5%, the small crystallites emerged between larger grains. Because the large and uniform grain size have been reported to by key for enhancement of the charge transport in the perovskites [13,21], the small amount of Rb incorporation could be expected to improve the photovoltaic and photodetector devices behaviors. The increase in the grain size by Rb doping was related to the stress-relief of the perovskite phase that incorporates the small ionic radius of Rb [19].

**Figure 2:**
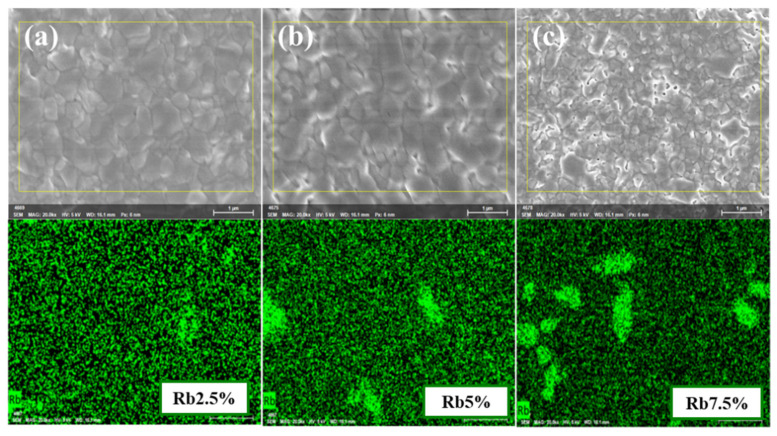
EDS mapping of Rb_x_(FA_0.75_MA_0.25_)_1-x_PbI_3_ with x = (**a**) 2.5% (**b**) 5% (**c**) 7.5% Rb, respectively.

**Figure 3:**
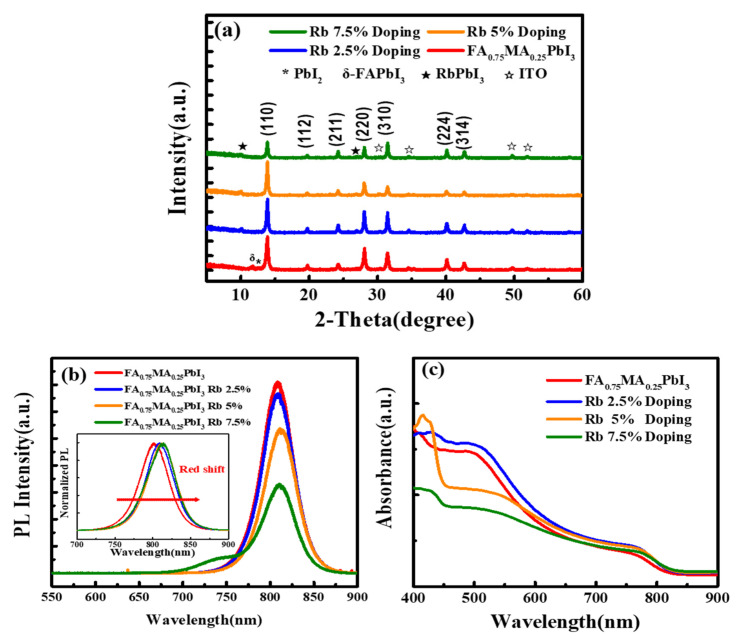
(**a**) XRD θ–2θ, (**b**) PL spectra, and (**c**) Absorbance of Rb_x_(FA_0.75_MA_0.25_)_1-x_PbI_3_ films with x = 0%, 2.5%, 5%, 7.5% Rb-doped, respectively.

The EDS elementary distribution of the Rb-doped films was shown in Figure 2 along with the clustering of Rb was found when the Rb concentration is higher than 2.5%, becoming more obvious when the doped Rb concentration reached 5% and 7.5%. The XRD patterns of the Rb_x_(FA_0.75_MA_0.25_)_1-x_PbI_3_ were shown in Figure 3. All of the diffraction peaks were associated with the planes of perovskites. The δ-FAPbI_3_ s phase was observed in the 0% Rb sample, but was absent when 2% Rb was added to the perovskite films. The photo-inactive yellow phase δ-FAPbI_3_ was almost eliminated by adding a moderate amount of Rb. Furthermore, PbI_2_ precipitation that usually precipitates at grain boundaries was also suppressed by 2% Rb doping. However, the RbPbI_3_ phase was found in the 5% and 7.5% Rb-doped samples that decreased the diffraction intensity. Figure 3b demonstrates the normalized PL peaks. The PL intensity increased by 2.5% Rb doping and the peaks red shift when the Rb content was increased, indicating a decrease in the band gap. This was ascribed to the Varshni shift that caused the band gap narrowing with the increase in the grain size [22]. Similar redshift upon RbI doping has been reported [23]. The PL intensity was slightly decreased by 2.5% Rb doping, but was markedly reduced by 5% and 7.5% Rb doping. This trend was similar with the variation of XRD intensity, therefore the mechanism was ascribed to the appearance of the yellow phase. Figure 3c shows the ultra-violet to red absorption spectra (400–650 nm) of the films. However, additional absorption edge was found around 430 nm for the 5% and 7.5% Rb doped samples. This is caused by the RbPbI_3_ that has a larger band gap (2.7 eV) than FA_0.75_MA_0.25_ PbI_3_ (~1.55 eV) [24]. Due to the Rb-rich clusters, the 7.5% Rb-doped perovskite film showed the worst absorbance. A red shift caused by Rb doping in FA_0.75_MA_0.25_PbI_3_ was obtained in the absorption spectrum, in good agreement with the PL result.

**Figure 4:**
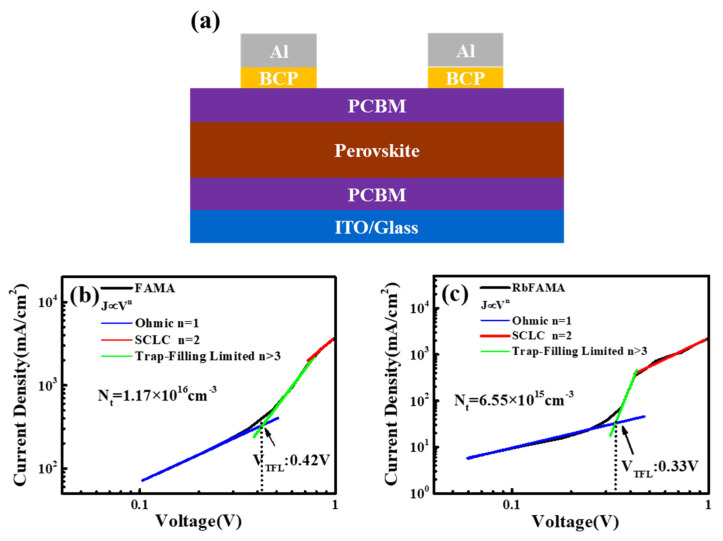
(**a**) SCLC device structure; (**b**) J-V relationship of FA_0.75_MA_0.25_PbI_3_, and; (**c**) J-V relationship of Rb_0.025_ FA_0.75_MA_0.25_PbI_3_. Trap-filling limit voltage (V_TFL_) was determined from the linear fitting in trap-filling region (green) and trap density (N_t_) was calculated accordingly.

In order to study the defect behavior for the Rb-doped perovskite, the SCLC device was fabricated and the density of defects and trap states can be calculated by the SCLC model. Figure 4 demonstrates the dark J-V curves, in which three regions with different slopes that corresponded to the linear ohmic region (J∝V^n^, *n* = 1) at low bias, trap-filling limited region (J∝V^n^, *n* > 3) at middle bias, and a SCLC or Chlid’s region (J∝V^n^, *n* = 2) at high bias were found and fitted linearly. The trap densities (N_t_) of the electron-only devices were calculated [21]. The trap-filling limited voltage V_TFL_ can be determined by the onset of trap-filling limited region, and then the N_t_ can be derived. The linear fitting of different regions was shown in the figure based on the equation, V_TFL_ = (q ∗ N_t_ ∗ d^2^)/(2 ∗ ε ∗ ε_0_), where q, N_t_, d, ε and ε_0_ are electronic charge, trap density, the thickness of device, the dielectric constant of perovskite and the permittivity of free space, respectively. The dielectric constant used here was derived by measuring parallel capacitance of the perovskite using the structure of Ag/silicon/SiO_2_/perovskite/Ag. The capacitance of native oxide Ag/silicon/SiO_2_/Ag was firstly measured as a reference. As a result, the Rb-doped films had a lower V_TFL_ of 0.33 V and trap densities 6.55 × 10^15^ cm^−3^ than FA_0.75_MA_0.25_PbI_3_ (0.42 V, 1.17 × 10^16^ cm^−3^).

**Figure 5:**
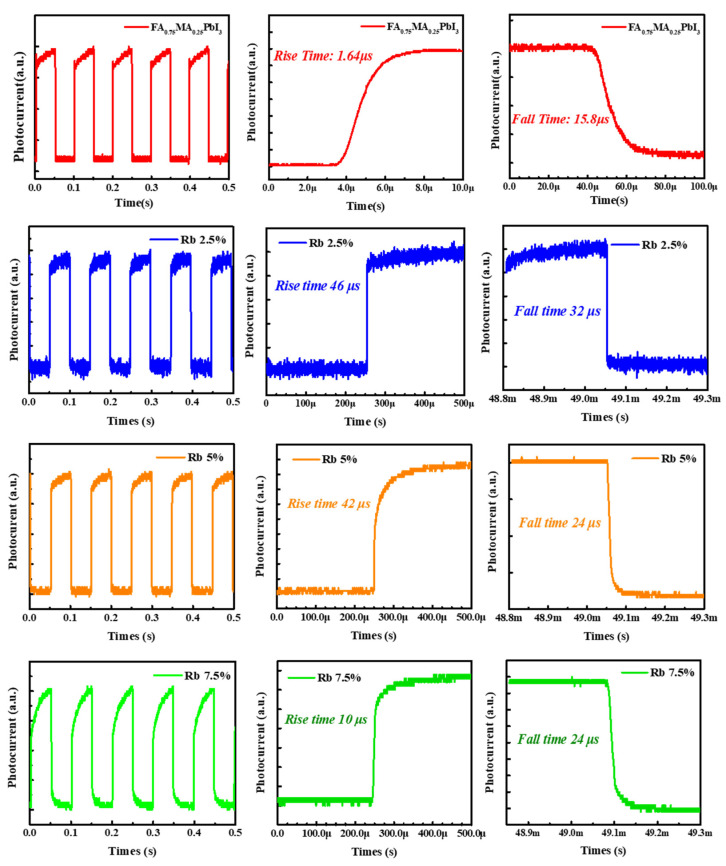
Cyclic photorespnse with time of perovskite FA_0.75_MA_0.25_PbI_3_ photodetectors with different Rb^+^ doping concentration of 0%, 2.5%, 5%, 7.5% Rb, and magnification in rise side and fall side, respectively.

Next, the influence of Rb^+^ doping concentration on the photodetector performance are discussed. The continuous light response was shown in Figure 5, and the periodic switching characteristics were clearly observed. A continuous light response at a microsecond speed was observed. The doping of Rb caused an increase in the response time. The reason is that the phase separation of Rb-rich phases leads to a decrease in the crystalline properties of the film, which hinders carrier transfer behaviors. Differently, the response time of 7.5% Rb-doped films was the lowest among all of the Rb-doped samples. It was ascribed to the increase in the grain size. According to the literature, the larger crystal grains have lower bulk defects and higher carrier mobility, speeding up the response time [25].

**Figure 6:**
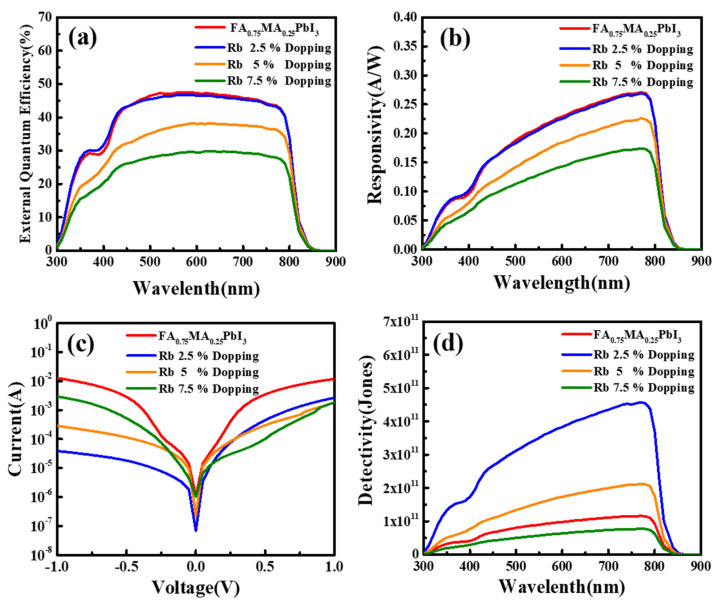
(**a**) EQE spectra, (**b**)responsivity, (**c**) I-V relationship without light, and (**d**) detectivity of perovskite PDs with various Rb contents.

Figure 6 shows the measured response time, spectral responsivity (R), noise current, EQE, and detectivity (D) of the PDs with various Rb. The EQE of the perovskite films was shown in Figure 6a. The 2.5% Rb-doped perovskite film had a highest EQE of ~50%. The enhancement of the EQE spectrum was caused by the increased absorption of the perovskite. The EQE spectra were further used to represent the R (Figure 6b) [26]. Because of the proportion relationship between EQE and R, the 2.5% Rb-doped perovskite film also demonstrated the highest R of 0.273A/W. Figure 6c demonstrated the dark current (I_D_) of the perovskite films. All of the Rb-doped showed lower leakage current compared with the undoped samples. Additionally, the 2.5% Rb-doped perovskite film had the lowest dark current. Detectivity of a PD can be determined from the I_D_ and R [26]. Figure 6d shows the relationship of D versus wavelengths. It was found that the detectivity was obviously increased by Rb doping for all of the Rb-doped films. Among these samples, the 2.5% Rb-doped PD showed the highest D (4.58 × 10^11^ Jones). The results revealed that the incorporation of Rb was mainly on the improvement of the detectivity, owing to the reduced internal defects and increased grain size and thin-film quality. Compared with the effect of Cs doping in the perovskite PDs, the Cs-doped PDs showed marked reduction in the response time but the change in the rise time and fall time of the Rb-doped PDs is not obvious [27]. The appearance of Rb-rich second phase should be responsible for this difference. 

The solar cell parameters were measured and shown in Figure S2 to further understand the photo-response mechanism of the Rb-doped PDs. The light source was an AM1.5G solar simulator. Similarly, 2.5% Rb^+^ doped solar cells had an increase in the conversion efficiency when it was compared with the undoped one, mainly caused by the great increase in the Jsc. It is believed that the improvement of the J_SC_ was related to the decline of the trap densities and improved crystal quality, caused by the incorporation of Rb that inhibited the formation of δ-FAPbI_3_ phases and internal defects.

## 4. Conclusions

The impacts of Rb doping on the organic perovskite PDs was investigated. It was found that slight addition of the Rb with 2.5% Rb increased the grain size, reducing the unwanted yellow phase. The lattice expansion was observed by XRD that shows monotonically shift of peaks toward higher angle when the Rb contents was increased. As a result, the red shift in PL and absorption spectra was observed by increasing Rb. SEM images shows that when the Rb concentration is higher than 2.5%, the Rb-rich cluster emerged. For the PD performance, the rise time and fall time changed little, but the detectivity was markedly enhanced owing to the reduction of the dark current by Rb doping. By SCLC analysis, the Rb doping was found to have passivated the defects in the perovskite, lowering the leakage current and reducing the trap densities of carriers.

## Data Availability

Data is contained within the article or supplementary material.

## Supplementary Materials

The following supporting information can be downloaded at: https://www.mdpi.com/article/10.3390/nano12010157/s1, Figure S1: Device structure of perovskite photodetectors. Figure S2: Parameters of solar cells of Rb-doped Rb_x_(FA_0.75_MA_0.25_PbI_3_)_1-x_ solar cells.
