# Supplementary material for: Properties of Halide Perovskite Photodetectors with Little Rubidium Incorporation
Source: Nanomaterials (Basel). 2022 Jan 3;12(1):157. doi: 10.3390/nano12010157 (PMC8746863; doi:10.3390/nano12010157)
Supplement: Supplementary file 1 [file nanomaterials-12-00157-s001.zip › nanomaterials-1504276-supplementary.pdf]

Supplementary Material

Properties of Halide Perovskite Photodetectors with Little Rubidium Incorporation

Yuan-Wen Hsiao [1], Jyun-You Song [1], Hsuan-Ta Wu [2], Ching-Chich Leu [3,*] and Chuan-Feng Shih [1,4,*]

1 Department of Electrical Engineering, National Cheng Kung University, Tainan 70101, Taiwan; n28064012@mail.ncku.edu.tw (Y.-W. H.); c2781130@gmail.com (J.-Y.S.)
2 Department and Institute of Electrical Engineering, Minghsin University of Science and Technology, Hsinchu 30401, Taiwan; htwu@must.edu.tw (H.-T.W.)
3 Department of Chemical and Materials Engineering, National University of Kaohsiung, Kaohsiung 81148, Taiwan.
4 Hierarchical Green-Energy Materials (Hi-GEM) Research Center, National Cheng Kung University, Tainan 70101, Taiwan.
* Correspondence: ccleu@nuk.edu.tw (C.-C.L.); cfshih@mail.ncku.edu.tw (C.-F.S.); Tel.: +886-7-5919456 (ext. 7456) (C.-C.L.); +886-6-2757575 (ext. 62398) (C.-F.S.)

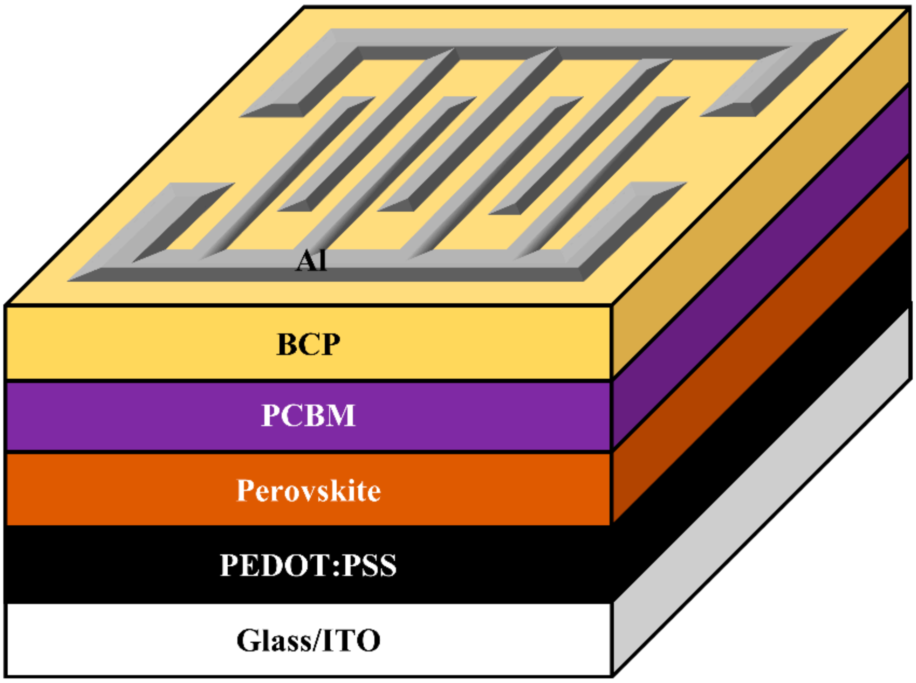

Figure S1. Device structure of perovskite photodetectors.

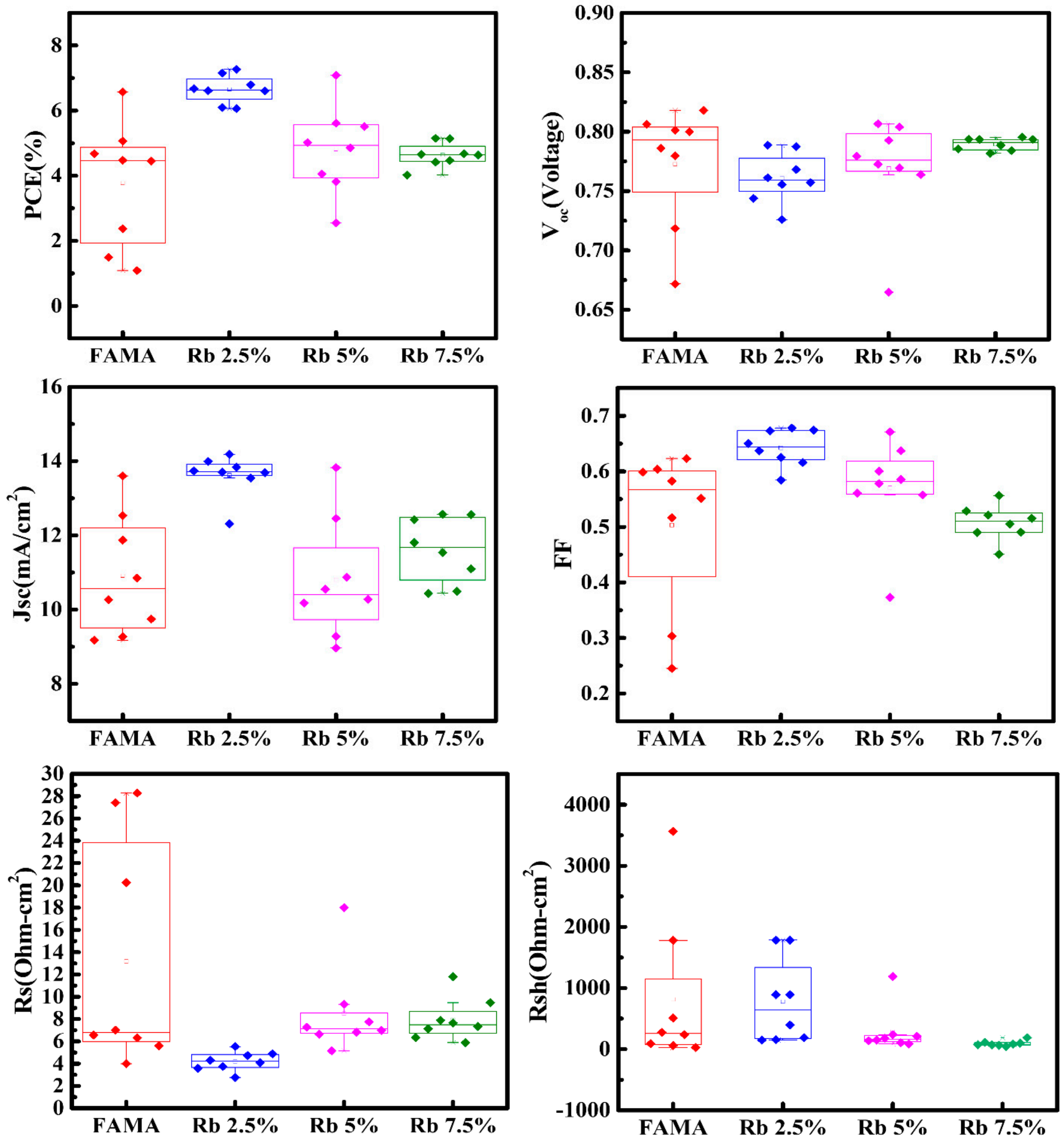

Figure S2. Parameters of solar cells of Rb-doped $Rb_x(FA_{0.75}MA_{0.25}PbI_3)_{1-x}$ solar cell.
